# Prenatal care among rural to urban migrant women in China

**DOI:** 10.1186/s12884-018-1934-7

**Published:** 2018-07-13

**Authors:** Zhanhong Zong, Jianyuan Huang, Xiaoming Sun, Jingshu Mao, Xingyu Shu, Norman Hearst

**Affiliations:** 1 School of Public Administration, Hohai University, Nanjing, China; 2 School of Sociology and Population Sciences, Nanjing University of Posts and Telecommunications, Nanjing, China; 3 Department of Family and Community Medicine, University of California, San Francisco, CA USA

**Keywords:** Prenatal care, Migration, Migrant women, China, Health insurance

## Abstract

**Background:**

There is a very large population of internal migrants in China, and the majority of migrant women are of childbearing age. Little is known about their utilization of prenatal care and factors that influence this. We examined this using data from a large national survey of migrants.

**Methods:**

5372 married rural to urban migrant women aged 20–34 who were included in the 2014 National Dynamic Monitoring Survey on Migrants and who delivered a baby within the previous two years were studied. We examined demographic and migration experience predictors of prenatal care in the first trimester and of adequate prenatal visits.

**Results:**

12.6% of migrant women reported no examination in the first trimester and 27.6% had less than 5 prenatal visits during their latest pregnancy. Multivariate analysis indicated that demographic predictors of delayed and inadequate care included lower educational level, lower income and not having childbearing insurance. Migrating before pregnancy, longer time since migration, having migrated a greater distance, and not returning to their home town for delivery were correlated with better prenatal care.

**Conclusions:**

Many internal migrant women in China do not receive adequate prenatal care. While internal migration before pregnancy seems to promote adequate prenatal care, it also creates barriers to receiving care. Strategies to improve prenatal care utilization include expanding access to childbearing insurance and timely education for women before and after they migrate.

## Background

In China, the speed of economic development has been rapid since the early 1980s, causing a rapid expansion in migration to industrialized urban areas. Between 1982 and 2015, the number of internal migrants is China is estimated to have increased from 6.5 million to 253 million [1–4]. Most migrants come from relatively poor and underdeveloped rural areas. Under China’s longstanding *hukou* (household registration) system, these migrants remain official residents of their communities of origin, a situation that can be an important institutional barrier to equal rights in terms of employment, education, housing, health care and social services, particularly for rural-to-urban migrants [5, 6]. The majority of internal migrants are young workers, and a large percentage of female internal migrants are of childbearing age [7]. Most people migrating from rural to urban areas do so to earn money and have little education, poor healthcare awareness, and limited ability to access health care [8].

Use of prenatal care is associated with reduced maternal mortality [9, 10], may reduce undesirable pregnancy outcomes such as low birthweight and preterm delivery [11], and provides women and their unborn children with access to various screening tests and interventions [12]. The World Health Organization recommends at least four prenatal visits with a detailed list of recommended components of antenatal care for developing and developed countries [13]. In China, prenatal care was regulated under the 1994 Law on Maternal and Infant Health, and these services are covered under the medical insurance system [14]. The recommendation is for at least five antenatal visits, beginning with at least one visit in the first trimester and two visits each in the second and third trimesters, with additional visits as needed [15, 16]. But uptake of healthcare services varies greatly between maternal populations. Absent or inadequate prenatal care was commonly reported among Chinese women in a study covering the years 1997 to 2015 [17].

Previous studies of migrant women have indicated that their utilization of prenatal care is lower than in the general population [18]. Compared to local residents, migrant women were substantially less likely to have prenatal examinations in one study (43.5% vs*.* 84.2% in Guangzhou [19]; 84.4% vs*.* 91.7% in Jiangsu province [20]). Another study in Shanghai showed that while 90.1% of migrant women had at least one prenatal care visit, only 49.7% had the five or more antenatal care visits recommended and only 19.7% visited an antenatal care center during the first trimester [16]. Other studies among migrant women of reproductive age showed that proportions receiving 5 or more prenatal examinations ranged from 37.6% in Beijing [21], 43.3% in Nanjing [22], and 72.3% in Wuhan to 75.5% in Jilin [23]. But such studies have been limited to specific samples in a few urban areas.

A few of these studies have also examined predictors of receiving inadequate prenatal care. Migrant women with younger age, lower educational level, and lower household income were less likely to have 5 or more prenatal examinations [16, 24]. Factors associated with higher likelihood of receiving adequate prenatal care include longer residency in the receiving community, first pregnancy, having medical insurance, husbands with higher educational level, and staying in the receiving community during pregnancy (as opposed to returning to their home town) [4, 20, 25, 26].

These previous studies of prenatal care among migrant women in China provide important insights, but they have limitations. Local studies may not necessarily be representative of China as a whole. Hospital-based studies of women delivering in urban areas exclude the large proportion of women who return to their home towns for pregnancy and delivery. Also, most studies have not measured important aspects of women’s migration experience, such as how long ago they migrated and from how far away. We therefore analyzed data from a large national survey of internal migrants in China conducted in 2014 to further examine these issues.

## Methods

### Setting

China has a longstanding household registration system under which official place of residence is based on place of birth and can only be permanently changed under certain limited circumstances with official permission. Internal migrants thus retain their official resident registration in their sending communities even after moving across administrative boundaries within or between municipal jurisdictions or between provinces. After leaving their homes for a month, they are officially considered migrants or “floaters.” No matter how long ago they moved, most internal migrants are not eligible for many public services, including government-provided health care, in their receiving communities since they don’t have official resident registration at the destination [27]. An important exception is family planning services, for which all married women of reproductive age are eligible, wherever they live. Health care is also readily available in urban areas from private or public providers, but this must be paid out of pocket without the medical insurance that local urban residents usually have.

Migrant women are eligible for rural health insurance in their sending communities (which requires them to actively enroll and pay nominal premiums). But rural health insurance does not directly cover services outside their home towns. In some circumstances, they can pay out-of-pocket for health care in their receiving communities and submit bills for reimbursement, but the process is cumbersome and reimbursement rates are incomplete and variable. They are not eligible for the urban health insurance that is available to people born in their receiving communities. The Chinese government has for many years had a stated goal of combining the rural and urban health insurance systems, but this has economic and administrative obstacles and there is no specific timeline for implementation.

Migrant women with full time official employment with a labor contract are eligible for separate childbearing insurance included in employee health insurance that covers both maternal medical care and salary for a three to four-month maternity leave and which is mostly paid by the employer, but this does not apply to the majority of female migrant workers who often have part time or temporary jobs or work in the informal sector [28]. Many migrant women stop working and return to their home towns when they are pregnant, typically from the second trimester through delivery [29].

### Data source

This study examined a subsample of the 2014 National Dynamic Monitoring Survey on Migrants. This large national survey has been conducted every year since 2009 by the China Population and Development Research Center of the National Health and Family Planning Commission (CPDRC). The Investigators applied for and were granted access to the data in 2014. This study could be considered a secondary data analysis in that none of the investigators were directly involved in data collection. The data that we received did not include any personal identifiers. All data were stored in password-protected form.

The Health and Family Planning Commissions of each province undertook the field work for this survey. This included providing basic data for CPDRC to compile the sampling frame, investigator training in the sampled cites, organizing field surveys, data input, data auditing and quality checks. The sampling frame was first initially compiled according to annual reports of internal migrants from each province in 2013 and then refined with new information provided by each province in April 2014. Overall coordination of the survey, including compiling the final sampling frame, sampling, questionnaire design, training of supervisory personnel, field supervision, quality control, data upload, and data management, was directed at the national level.

The total sample was chosen with a stratified, multi-stage and Probability Proportional to Size (PPS) sampling method. Survey information was collected by structured face-to-face interview. Domains included demographic characteristics, employment and family income and expenditure, public health and medical service utilization, childbearing, and family planning service utilization among internal immigrants, both men and women, aged 15–59 throughout China in May 2014. Each of 31 provinces and 1 provincial management unit (XPCC), were regarded as strata. The sample had three stages (sub-district/town, neighborhood or village committee, and individual) and was proportional to the size of the migrant population in each. The sample size by province was also roughly proportional to the size of the migrant population: 14000 interviewees in Zhejiang province, 12,000 in Jiangsu and Guangdong, 10,000 in Heilongjiang, 8000 in Beijing and Shanghai, 7000 in Fujian and Hunan, 6000 in 9 provinces including Tianjin and Shandong, 5000 in 9 provinces including Hebei and Shanxi, and 4000 in Jilin, Guizhou, Tibet, Ningxia, Xinjiang and XPCC. According to the survey plan, 201,000 interviewees were to be interviewed; the final sample size was 200,937.

Supervisors in charge of quality control and training at the provincial level were trained at the national level. Uniform survey manuals and investigator handbooks were printed and distributed to each province by CPDRC. In the field, investigators visited the sampled migrants and interviewed them for the survey. Each interview took about 20–30 min. Potential participants received a printed information sheet explaining the study and informing them that participation is voluntary. They then either declined or gave verbal consent to participate. Refusals were rare (as is common for such surveys in China) and were not tabulated; sampled individuals who did not participate were replaced. Completed questionnaires were checked for quality by supervisors. Each province scheduled and finished the survey in May 2014. Further details of the overall survey methodology are available in the “2015 Report on China’s Migrant Population Development.” [30]

This study was conducted on a subset of the survey described above. Criteria included a rural *Hukou* (household registration), female, married, aged 20–34, and having delivered a baby since January of 2012. This subset thus included women giving birth at ages as young as 17 years 8 months and accounted for 93.6% of all women in the larger survey from rural *Hukous* who gave birth in the previous two years. The resulting sample size was 5372. Prenatal care utilization data were considered for the youngest child if a respondent had more than one delivery since 2012.

### Dependent variables

Regarding prenatal care utilization, there were 2 dependent variables considered. The first was a yes or no question as to whether a respondent received an examination in the first trimester of this pregnancy with an answer of not remembering being considered as a missing value. The second was whether or not the woman had at least 5 prenatal visits. Respondents were asked how many total visits for prenatal examinations they had during this pregnancy, and we dichotomized this as 5 or more (the minimum standard for adequate prenatal care in China) or less than 5.

### Independent variables

Two main groups of independent variables were analyzed: those related to demographic characteristics and those related to migration characteristics. For the demographic characteristics, age was divided into 3 categories: 20–24 years, 25–29 years, and 30–34 years; educational level was asked with 7 options (illiterate, elementary, middle school, high school, junior college, undergraduate, graduate) and was recoded into 4 categories: elementary school, middle school, high school, or college; area of origin was recoded into 3 categories: east, central, and west (these are recognized regions of China with substantial differences in development); birth order of this child was coded 1st, 2nd, and 3rd or more (only 17 migrant women had 4 children and one woman had 5); having medical insurance was recoded into 4 categories: rural medical insurance, childbearing insurance, both insurances, and no insurance; household monthly income per capita (CNY) was divided into 4 categories: < 1000, 1000–2000, 2000–3000, and > =3000. For migration characteristics, migration before this pregnancy was coded yes or no (some women did not first migrate until after their recent pregnancy); geographic spread of migration was recoded into 3 categories: between provinces (from a province of origin to another province), between municipal jurisdictions within province (from a city of origin to another city in a province), and within municipal jurisdiction (municipal jurisdictions in China often include large surrounding rural areas.) Staying at one’s rural hometown during pregnancy was coded yes or no. Time since first migration (in years) was a numerical variable calculated with 2 time variables: time of first migration and time of the survey.

### Data analysis

Data were analyzed with SPSS 20.0. The bivariate associations between prenatal examinations and demographic and migration characteristics were examined using the chi-square test and the F test for the continuous variable of time since migration. We assessed independent predictors of dependent variables with forward stepwise logistic regression that retained predictors with *p* < 0.05 in the final multivariate model. Women who had not yet migrated at the time of their pregnancy were excluded from analyses that included variables that did not apply to them at the time they were pregnant: geographic spread of migration, whether they stayed at their home town during pregnancy, and time since migration.

## Results

### Sample composition

The sample consisted of 5372 married rural-to-urban migrant women, 48.6% of whom were aged 25–29 years, 92.6% had at least a middle school education, 75.8% migrated from central or west China, 72.7% had only rural medical insurance, 78.8% had migrated before this latest pregnancy, and 50.2% had monthly household per capita income of 1000–2000 CNY (Table 1). Among these migrant women, 37.5% had older children, of whom 93.1% had one child previously and 63.8% had a girl. Regarding specific migration characteristics, nearly half of migrant women (48%) moved from one province to another, and 23.7% went back and stayed at their rural home town during pregnancy. The average time since first migration was 5 years (standard deviation (SD) = 4), with a maximum of 26.

**Table 1:** Sample Characteristics (*N* = 5372)

Characteristics	n(%)
Age group	
20–24	1554(28.9)
25–29	2608(48.6)
30–34	1210(22.5)
Educational level	
Elementary	395(7.4)
Middle school	3025(56.3)
High school	1228(22.9)
College	724(13.5)
Area of origin	
East	1311(24.4)
Central	2171(40.4)
West	1890(35.2)
Monthly household income per capita(CNY)^a^	
< 1000	995(18.5)
1000–2000	2697(50.2)
2000–3000	923(17.2)
≥ 3000	757(14.1)
Medical insurance	
Rural insurance	3908(72.7)
Childbearing insurance	487(9.1)
Both	131(2.4)
None	846(15.7)
Birth order of this child	
1st	3358(62.5)
2nd	1874(34.9)
3rd	140(2.6)
Migrated before this pregnancy	
Yes	4233(78.8)
No	1139(21.2)
Geographic spread of migration	
Between provinces	2565(47.7)
Between municipal jurisdictions within province	1728(32.2)
Within municipal jurisdiction (rural to urban)	1079(20.1)
Stayed at home town during pregnancy	
Yes	1274(23.7)
No	4098(76.3)
Time since first migration (years)	
mean ± SD	5 ± 4

^a^1 US dollar is approximately 6.5 CNY

### Prenatal care utilization in the first trimester

Regarding initiation of prenatal care, 12.6% of migrant women reported they had no examination in the first trimester of pregnancy, and 3% could not remember this information clearly. Among 5213 respondents with complete data, bivariate predictors of not initiating prenatal care in the first trimester included age, although differences by age group were small (as presented in Table 2.) Stronger predictors of delayed initiation of prenatal care included lower educational level, central and west region of origin, lower monthly per capita household income, lack of insurance, and higher birth order. In the multivariate logistic model, only 4 variables were independent predictors of late initiation of prenatal care. These included lower educational level (elementary school, odds ratio [OR] = 1.98, confidence interval [CI] = 1.31 to 3.01; middle school, OR = 1.50, CI = 1.08 to 2.10; high school, OR = 1.18, CI = 0.82 to 1.70; index category, college), region of origin (east region, OR = 0.68, CI = 0.53 to 0.86; central region, OR = 1.10, CI = 0.92 to 1.34; index category, west region), birth order (the third child, OR = 3.17, CI = 2.13 to 4.74; the second child, OR = 1.78, CI = 1.49 to 2.12; index category, the first child), and medical insurance (childbearing insurance, OR = 0.61, CI = 0.39 to 0.97; rural medical insurance, OR = 1.11, CI = 0.88 to 1.39; both, OR = 0.67, CI = 0.33 to 1.38; index category, no insurance.) Variables not significantly associated with late initiation of prenatal care in the multivariate model included age group, household income, and migration before pregnancy.

**Table 2:** Bivariate predictors of low prenatal care utilization

	None in 1st trimester, *N* = 5213	Less than 5 visits, *N* = 5372
Characteristics	n(%)	n(%)
Age group	*p* = 0.04	*p* = 0.70
20–24	189(12.5)	434(27.9)
25–29	295(11.6)	705(27.0)
30–34	171(14.6)	341(28.2)
Educational level	*p* = 0.000	*p* = 0.000
Elementary	78(20.5)	182(46.1)
Middle school	415(14.2)	888(29.4)
High school	116(9.7)	291(23.7)
College	46(6.5)	119(16.4)
Area of origin	*p* = 0.000	*p* = 0.000
East	106(8.2)	233(17.8)
Central	302(14.3)	644(29.7)
West	247(13.6)	603(31.9)
Household income per capita(CNY)	*p* = 0.001	p = 0.000
< 1000	148(15.4)	363(36.5)
1000–2000	342(13.1)	738(27.4)
2000–3000	85(9.5)	226(24.5)
≥3000	80(10.8)	153(20.2)
Medical insurance	*p* = 0.000	*P* = 0.000
Rural insurance	517(13.7)	1173(30.0)
Childbearing insurance insurance	27(5.6)	63(12.9)
Both	9(7.0)	22(16.8)
None	102(12.3)	222(26.2)
Birth order pregnancy	*p* = 0.000	*P* = 0.000
1st	303(9.3)	820(24.4)
2nd	313(17.2)	584(31.2)
3rd	39(28.7)	76(54.3)
Migrated before his pregnancy	*p* = 0.91	p = 0.000
Yes	517(12.5)	1076(25.4)
No	138(12.7)	404(35.5)
Geographic spread of migration^a^	*P* = 0.002	p = 0.000
Between provinces	277(14.0)	509(25.1)
Between municipal jurisdictions within province	136(10.0)	317(22.9)
Within municipal jurisdiction (rural to urban)	104(13.2)	250(30.5)
Stayed at home town during pregnancy^a^	*P* = 0.06	*p* = 0.003
Yes	78(15.2)	165(30.6)
No	439(12.2)	911(24.7)
Time since first migration(years)^a^	*P* = 0.26	*p* = 0.09
Eta correlation	0.01	0.1

^a^Includes only women who migrated before pregnancy (*N* = 4233)

### Adequate number of prenatal examinations

Among all respondents, the number of prenatal examinations ranged from 1 to 28 with a mean of 7 and standard deviation [SD] of 3. 72.4% of migrant women had 5 or more examinations during pregnancy, 42.9% had 8 or more, and 10.4% had at least 12. But 27.6% of migrant women had less than the 5 examinations considered to be the minimum standard in China, and 12.3% had 3 or fewer examinations.

Bivariate predictors of an inadequate number of prenatal examinations included educational level, area of origin, monthly household income, medical insurance, birth order and migration before pregnancy (Table 2.) The average number of prenatal examinations among those migrating before pregnancy was 7 compared to 6 among those migrating after. Table 3 shows significant independent predictors of an inadequate number of prenatal examinations. These included lower educational level, origin in central and west China, higher birth order, not having childbearing insurance, and lower per capita household income. Women who had not yet migrated at the time of their pregnancy were more likely to have an inadequate number of prenatal examinations.

**Table 3:** Multivariate predicators of low prenatal care utilization

	Model I		Model II^a^	
	Less than 5 visits,N = 5372		Less than 5 visits, N = 4233	
Characteristics	OR(95% c.i.)	*p*	OR(95% c.i.)	*p*
Educational level		< 0.001		< 0.001
Elementary	2.38(1.76,3.22)	< 0.001	3.00(2.14,4.21)	< 0.001
Middle school	1.39(1.10,1.74)	0.005	1.39(1.08,1.79)	0.012
High school	1.22(0.95,1.55)	0.12	1.18(0.90,1.56)	0.23
College	index			
Area of origin		< 0.001		< 0.001
East	0.58(0.48,0.69)	< 0.001	0.54(0.44,0.67)	< 0.001
Central	0.99(0.86,1.13)	0.83	1.00(0.85,1.17)	0.98
West	index			
Birth order		< 0.001		< 0.001
3rd	1.18(1.03,1.35)	0.015	1.42(1.21,1.66)	< 0.001
2nd	2.80(1.96,3.99)	< 0.001	3.50(2.32,5.26)	< 0.001
1st	index			
Medical insurance		< 0.001		0.002
Rural insurance	1.18(0.99,1.40)	0.062	1.12(0.91,1.36)	0.29
Childbearing insurance	0.62(0.45,0.86)	0.004	0.60(0.42,0.85)	0.005
Both	0.74(0.45,1.21)	0.23	0.86(0.51,1.47)	0.63
None	index			
Household income		0.004		
1000-	1.52(1.21,1.52)	< 0.001		
1000–2000	1.23(1.01,1.51)	0.044		
2000–3000	1.23(0.97,1.56)	0.086		
3000+	index			
Migrated before this pregnancy		< 0.001		
No	1.52(1.31,1.75)			
Yes	index			
Geographic spread of migration				0.005
Between provinces			0.73(0.61,0.89)	0.001
Between municipal jurisdictions within province			0.78(0.63,0.95)	0.016
Within municipal jurisdiction (rural to urban)			index	
Stayed at home town during pregnancy				0.008
Yes			1.32(1.08,1.62)	
No			index	
Time since first migration(years)			0.98(0.96,0.99)	0.005

^a^Includes only women who migrated before pregnancy (N = 4233)

Several variables related to migration experience were significant predictors of an inadequate number of prenatal visits in the multivariate models (Table 3). In model I (including all women), those who migrated after pregnancy were more likely to have an inadequate number of prenatal visits. In model 2 (excluding women who had not yet migrated at the time of pregnancy), women migrating from less distant areas, women who migrated more recently, and women who returned to their home towns during pregnancy were more likely to have an inadequate number of prenatal visits.

## Discussion

These results regarding prenatal care from a large national survey of migrant women show that a substantial minority of migrant women in China receive delayed or inadequate prenatal care and suggest that utilization of prenatal care is influenced by a combination of demand and access factors. The strongest predictors seem to be related to demand, and this seems mainly related to assimilation in their new urban environment. Women who migrated before they became pregnant, who migrated longer ago, and who did not return to their home towns during pregnancy were more likely to begin prenatal care in the first trimester and/or to have an adequate number of prenatal visits. This was observed despite the fact that the great majority of these women did not have adequate insurance coverage for prenatal care in their new urban homes.

Nevertheless, access and insurance also appear to be important, as indicated by the higher rate of early initiation of prenatal care and higher number or prenatal visits for the minority of women with childbearing insurance. It is interesting that having rural insurance tended to *decrease* utilization of prenatal care. While this insurance has promoted health care access for rural people and might improve their economic access [31], it may also be a marker for closer ties to rural culture and less assimilation into migrants’ new urban homes. On the other hand, having childbearing insurance (with or without rural insurance) was associated with better utilization of prenatal care, probably because it both improved economic access and also may have been a marker for assimilation in the urban environment.

This study using data from a national survey of internal migrants found that a substantial minority of pregnant migrant women aged 20–34 in China receive either delayed prenatal care or less than the minimum recommended number of examinations: 12.6 and 27.6% respectively. Whether these percentages are “high” or “low” depends on the comparison group. Other studies indicate that these percentages are lower than for rural women in sending communities, but higher than for natives of the cities where these women now live [20, 26, 32]. Our results indicate that women who were migrants when they became pregnant were more likely to receive timely and adequate prenatal care than similar women who migrated after their pregnancy. These findings are consistent with the substantial regional differences in most health and development indicators that currently exist in China.

Migration is usually driven by economic factors initially, and it creates challenges in accessing medical care due to lack of official local residence and medical insurance. On the other hand, it also appears to bring advantages. Migrant women appear to move toward the prenatal care utilization pattern of native women in their new homes, and this becomes more the case the longer they stay and the more assimilated they become (for example, not returning to their home town for delivery and not maintaining rural health insurance in their communities of origin.) This influence appears stronger than the negative effect of barriers to care that migrants face.

Nevertheless, economic factors remain important, as demonstrated by the much higher rates of early and adequate prenatal care among women with childbearing insurance. Findings from other studies about demographic predictors of inadequate prenatal care are consistent with the results of this study regarding lower educational level, lower household income, and the latter birth order of the child [16, 19]. Adequate prenatal care is important for realizing China’s health goals for maternal and child health adopted in 2016. These include reducing maternal mortality rate from 20.1/100,000 in 2015 to 18/100,000 in 2020 and reducing infant mortality from 8.1/1000 in 2015 to 7.5/1000 in 2020 and 5.0/1000 in 2030 [33]. In 2010, maternal mortality rates in the mainly rural central and western regions of China (29.1 and 45.1 per 100,000 respectively) were higher than that in the eastern region (17.8 per 100,000) [34]. High maternal mortality rates have also been found among rural-to-urban migrant women in some cities [35]. Maternal health has become one of the three major health priorities for migrants, the other two being infectious diseases and occupational diseases and injuries [7].

International studies in both developing countries and developed countries also concluded that migrant women were more likely to have inadequate prenatal care, with some of the same socioeconomic predictors as found in this study, including lower education, lower income and lack of insurance [13, 36]. One study in Malaysia found that while few migrant women never received any prenatal care during their pregnancy, they tended to initiate prenatal care as late as 7 months compared to local citizens starting prenatal care early in the first trimester [37]. Another study of migrant women in India indicated that only 37% of rural to urban migrants had adequate prenatal care and that recent migrants had prenatal visits significantly less than those who were more settled (35% VS. 39%) [38].

This study has many limitations. It did not include a comparison group of non migrant women for direct comparison. Cross-sectional surveys only measure association and cannot prove cause and effect. There may have been recall bias when respondents reported their prenatal care experience; indeed, 3% of women said that they could not remember if they had care in their first trimester. Although this study was based on a national survey, it is possible that participants were not fully representative of all migrant women in China. The questionnaire may not have captured all aspects of the migration experience and other important confounding variables.

## Conclusion

Many young rural-to-urban migrant women report no prenatal care in the first trimester of pregnancy and an inadequate number of prenatal visits during pregnancy. Internal migration before pregnancy seems to promote adequate prenatal care compared to women who remain in rural areas, but it also creates barriers to access of care, and migrant women receive less care than native women in their new cities. Migrant women who are less educated, who have lower income, who do not have childbearing insurance, and who migrate from more nearby rural areas are more likely to have inadequate prenatal care suggesting strategies for targeting services.

Our results suggest several strategies that might increase prenatal care for migrant women. These include increasing access for migrant women to childbearing insurance and/or other medical insurance to cover prenatal care in their new homes. Efforts to coordinate services for the large number of women who return to their home towns for delivery might encourage these women to initiate care earlier (before returning home) and to receive more care overall. Education about the importance of prenatal care is also key. Ideally, this should take place in rural areas before these women migrate and by middle school at the latest. Such efforts would benefit not only women who migrate but also those who remain in rural areas.

## Abbreviation

CPDRC: China Population and Development Research Center of the National Health and Family Planning Commission
